# Non-Alcoholic Fatty Liver Disease (NAFLD) and Bariatric/Metabolic Surgery as Its Treatment Option: A Review

**DOI:** 10.3390/jcm10245721

**Published:** 2021-12-07

**Authors:** Paulina Głuszyńska, Dorota Lemancewicz, Janusz Bogdan Dzięcioł, Hady Razak Hady

**Affiliations:** 1 Department of General and Endocrine Surgery, Medical University of Bialystok, 15-089 Białystok, Poland; hadyrazakh@wp.pl; 2 Department of Human Anatomy, Medical University of Bialystok, 15-089 Białystok, Poland; dorota.lemancewicz@umb.edu.pl (D.L.); anatomia@umb.edu.pl (J.B.D.)

**Keywords:** non-alcoholic fatty liver disease, non-alcoholic steatohepatitis, obesity, bariatric surgery, laparoscopic sleeve gastrectomy, Roux-en-Y gastric bypass

## Abstract

The prevalence of non-alcoholic fatty liver disease (NAFLD) and non-alcoholic steatohepatitis (NASH) has considerably increased over the last years. NAFLD is currently the most common cause of chronic liver disease in the developing world. The diagnosis of NAFLD/NASH is often incidental, as the early-stage of disease is frequently free of symptoms. Most patients recognized with NAFLD have severe obesity and other obesity-related disease such as type 2 diabetes mellitus (T2DM), insulin-resistance, dyslipidemia and hypertension. The only proven method for NAFLD improvement and resolution is weight loss. Bariatric surgery leads to significant and long-term weight loss as well as improvement of coexisting diseases. There is a lot of evidence suggesting that metabolic/bariatric surgery is an effective method of NAFLD treatment that leads to reduction in steatosis, hepatic inflammation and fibrosis. However, there is still a need to perform long-term studies in order to determine the role of bariatric surgery as a treatment option for NAFLD and NASH. This review discusses current evidence about epidemiology, pathogenesis and treatment options for NAFLD including bariatric/metabolic surgery and its effect on improvement and resolution of NAFLD.

## 1. Introduction

Unhealthy lifestyle and dietary habits have contributed to an alarming increase in obesity and obesity-related diseases worldwide. The epidemic of obesity has led to a significant increase in the prevalence of non-alcoholic fatty liver disease (NAFLD). The prevalence of NAFLD is 25–30% of the general population and 50–90% in patients with obesity [1,2]. A recent report estimates the constant increase in the prevalence of NAFLD by the year 2030 with significant rise in hepatocellular carcinoma (HCC) and liver-related deaths [3]. NAFLD is the initial, uncomplicated medical condition that may lead to end-stage liver disease from non-alcoholic simple steatosis and steatohepatitis (NASH) to fibrosis and liver cirrhosis with its clinical consequences such as: variceal bleeding, ascites, renal failure, encephalopathy and spontaneous bacterial peritonitis [4,5]. Data from the European Liver Transplant Registry (ELTR) and United Network for Organ Sharing (UNOS) show that NAFLD and NASH have been the most rapidly growing indication for liver transplant within the last 20 years. Additionally, NAFLD is presently the most frequent non-viral hepatitis-related indication for liver transplant among adults in the United States [6,7].

NAFLD is frequently recognized as the hepatic manifestation of metabolic syndrome (MS) and remains in close association with components of MS that include increased fasting plasma glucose level and type 2 diabetes mellitus (T2DM), increased waist circumference, hypertension and dyslipidemia [8,9]. Recent studies have shown that over 80% of patients undergoing bariatric surgery have been diagnosed with NAFLD or NASH [10,11].

Bariatric/metabolic surgery is an effective treatment for morbid obesity that provides sustained and considerable weight loss with the improvement of obesity-related diseases. Reduction in body weight induced by bariatric surgery leads to potential decrease in hepatic inflammation, fat accumulation and fibrosis [12]. In the forthcoming sections of this review, we provide the information about pathogenesis, diagnosis and potential treatment options including conservative, pharmacological and bariatric surgery procedures for NAFLD according to the available literature.

## 2. Epidemiology

A systematic review conducted by Younossi et al. estimated the pooled, overall global prevalence of NAFLD diagnosed by imaging to be 25.24% (95% confidence interval (CI): 22.10–28.65). Their study reported the highest prevalence of NAFLD in South America (30.4%) and the Middle East (31.8%), whereas the lowest rate was reported in Africa (13.5%). The prevalences of NAFLD among patients diagnosed by blood test were 13.00% (95% CI: 4.44–32.47) for Europe, 12.89% (95% CI: 8.32–19.44) for North America, and 9.26% (95% CI: 7.07–12.05) for Asia [13]. According to Cholangitas et al., pooled NAFLD prevalence was 26.9% in the adult European population. Pooled NAFLD prevalence was higher in men than in women (32.8% vs. 19.6%). There were no differences between Mediterranean and non-Mediterranean countries. The pooled prevalence of NAFLD was higher in studies using ultrasonography and fatty liver index (FLI) for NAFLD diagnosis (27.2% and 30.1%, respectively) [14]. Current trends in dietary habits and preponderance of sedentary lifestyle contribute to the constant growth in the incidence of NAFLD worldwide. The National Health and Nutrition Examination Surveys data demonstrated a rise in the prevalence of NAFLD in the US from 5.5% (1988–1994) to 11% (2005–2008) [8], as it is estimated that the epidemic of obesity will continue to fuel the burden of NAFLD.

## 3. Pathogenesis of NAFLD

The pathogenesis of NAFLD is multifactorial; however, its understanding is crucial for the proper therapeutic interventions. A two-hit model of NAFLD development was proposed with the first hit consisting of hepatic steatosis, which then sensitizes the liver to injury mediated by “second hits” including: inflammatory cytokines, adipokines and oxidative stress leading to steatohepatitis and fibrosis [15]. This two-hit model has lost some favor, as it turned out too simplistic to fully describe the evolution of NAFLD, as different factors affecting disease development and progression were unveiled. Nowadays, the two-hit hypothesis was replaced with the “multiple hit” theory, which recognizes the following components in NAFLD pathophysiology: insulin resistance, obesity, gut microbiota, environmental and genetic factors. The key concept of NAFLD pathogenesis is excessive triglycerides hepatic accumulation as a result of imbalance between free fatty acids influx and efflux [16]. Excessive hepatic fat accumulation occurs in patients with obesity and T2DM, who have impaired insulin signaling. Insulin resistance leads to an uncontrolled lipolysis in adipose tissue that results in significant deposition of nonesterified free fatty acids (NFFA) in the liver [17]. Other factors contributing to excessive hepatic fat accumulation are dietary fats and de novo lipogenesis. Among dietary factors, fructose seems to have an important role, as it is both a substrate and an inducer for de novo hepatic lipogenesis [18]. The excessive inflow of triglycerides to the liver leads to inflammation, reactive oxygen species (ROS) formation, hepatocyte impaired function and lipotoxicity. Hepatocellular cells injury activates apoptotic pathways causing cellular death. This results in the progression from noninflammatory isolated steatosis to the development of nonalcoholic steatohepatitis with a risk of further evolution to fibrosis, cirrhosis and at worst to the development of hepatocellular carcinoma [19,20].

Available research shows that gut microbiota is also associated with the development of NAFLD and NASH [21,22]. The imbalance between protective and harmful bacteria, damage of intestinal barrier and disturbed immune response cause that bacterial products reach the liver through the portal vein and activate pathways responsible for proinflammatory response. Additionally, microbiota dysbiosis increases lipoprotein lipase activity and triglycerides accumulation by either decreasing choline levels or increasing methylamine level, which promotes development of NAFLD [23]. Damage of intestinal epithelial membrane leads to an impaired transport across the mucosa. Rahman et al. proved that compromised intestinal epithelial permeability contributes to development of NAFLD. The above-mentioned study showed that mice with defects or loss of junctional adhesion molecule A (JAM-A) in intestinal epithelial membrane develop more severe steatohepatitis after a diet high in saturated fat, fructose and cholesterol for 8 weeks. They also found out that colon tissue from patients with NAFLD has lower level of JAM-A and higher inflammation status as compared to patients without NAFLD [24]. Significant changes in gut microbiota are reported after bariatric surgery. Possible mechanisms for the intestinal microbiota changes include reduction in body weight, changes in food consumption, changes in ghrelin and leptin secretion and alternations in stomach pH [25,26].

**Figure 1:**
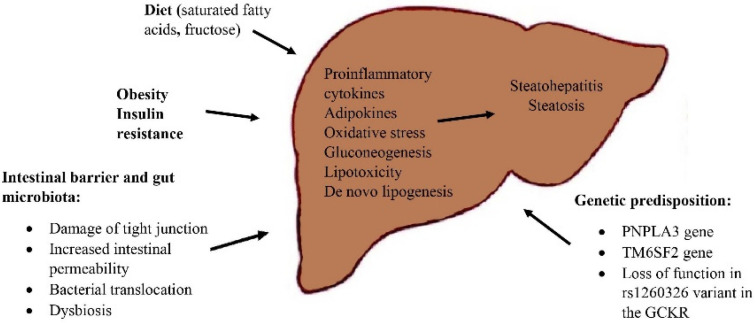
Pathogenesis of NAFLD.

Genes also have a role in the development of NAFLD. It has been discovered that genetic polymorphism can influence the NAFLD development and progression by variability in oxidative stress, inflammation and FFAs accumulation. The main genetic determinant of interindividual differences in hepatic fat content is nonsynonymous variant of patatin-like phospholipase 3 (*PNPLA3*) gene (rs738409 C/G, I148M), also known as adiponutrion [27]. The PNPLA3 variant has impaired hydrolysis activity and is less available for degradation, which leads to retention in of TG and polyunsaturated fatty acids priming accumulation of hepatic fat [28]. Another relevant genetic variant related to progressive NAFLD is the transmembrane 6 superfamily member 2 (*TM6SF2*), which is responsible for lipid retention and impairment of very low-density lipoprotein (VLDL) release by liver [29]. Loss of function in rs1260326 variant in the *GCKR* gene is also associated with increased TG concentration, steatosis and liver damage [30]. The understanding of possible nutrigenomic approaches may lead to improvement of NAFLD management and introduction of proper therapeutic strategy Figure 1.

## 4. Diagnosis of NAFLD

NAFLD is defined as an excessive accumulation of triglycerides in hepatocytes either by imaging or histology, simultaneously with exclusion of any significant alcohol consumption and other liver diseases [31]. Mildly elevated serum aminotransferases are the primary abnormality in NASH, although they may remain at normal level in up to 80% of patients. The alanine transaminase (ALT) level is generally higher than that of aspartate aminotransferase (AST). Other common findings in blood examination include high serum triglyceride and low HDL cholesterol level. With the development of the disease hypoalbuminemia, hyperbilirubinemia and thrombocytopenia may occur due to progression of liver injury [32]. Ultrasound is a non-invasive and widely available tool for the diagnosis of NAFLD. Characteristic sonographic findings for NAFLD include heterogeneity of liver; thick subcutaneous depth (>2 cm); quick attenuation of image 4–5 cm of depth, making deeper structures difficult to decipher, and; dispersion of echogenicity [33]. However, the use of ultrasound is very limited in patients with overweight and obesity due to excessive subcutaneous fat accumulation. The assessment of liver fibrosis without histological examination can be made by a combination of serological and imaging tests. There are several scoring systems used to estimate liver fibrosis without performing liver biopsy. NAFLD fibrosis score (NFS) is calculated based on following measurements: age, BMI, glucose blood concentration, platelet count, albumin serum level and AST/ALT ratio. Another one is the BARD score, which is composed of 3 variables: ALT/AST ratio, BMI and the presence of diabetes. BARD score of 0 or 1 are of high (96%) negative predictive value (NPV) for advanced fibrosis [34]. The AASSLD guidelines suggest the use of NFS or APRI score as non-invasive tools for clinical diagnosis. It is worth mentioning that NFS was developed as a scoring system for usage in patients with NAFLD [35]. The available ways to estimate liver fibrosis together with measured parameters are listed in Table 1 [36].

Magnetic resonance imaging (MRI) is a non-invasive method widely accepted by patients and doctors, and may be used as an alternative to liver biopsy in assessment of hepatic fat content [37]. Several studies have shown that magnetic resonance elastography (MRE) is a diagnostic tool for prediction of hepatic fibrosis stage in NAFLD with sensitivity of 63–87%, and specificity of 81–95% [38]. Another tool is magnetic resonance imaging-proton density fat fraction (MRI-PDFF), which has high accuracy in detecting hepatic steatosis and quantifying the degree of steatosis in NAFLD [39]. However, the gold standard for NAFLD diagnosis remains the percutaneous liver biopsy. Although liver biopsy is expensive, has increased risk of adverse events and requires professional interpretation, it should be performed in patients who benefit the most from making the right diagnosis.

According to the American Association of Liver Disease (AASLD), liver biopsy should be considered in patients with NAFLD who are at higher risk of steatohepatitis and advanced fibrosis, including those with diabetes and/or metabolic syndrome. Referral for liver biopsy should be also considered in patients who have findings of concern for cirrhosis, such as hypoalbuminemia, thrombocytopenia, AST > ALT and in patients undergoing cholecystectomy or bariatric surgery, when intraoperative biopsy is a low risk procedure [40]. The main histological characteristics of NAFLD is the accumulation of fat in the form of triglycerides within hepatocytes. The presence of >5% steatotic hepatocytes in a liver tissue is the criteria for the histological definition of NAFLD. In NAFLD, steatosis is usually macrovesicular, which means that lipid vacuole fills nearly the whole hepatocyte, and the nucleus is pushed to the side. A simple four-point scoring system that takes into account only macro- and/or mediovesicualar steatosis and estimates the percentage of hepatocytes covered with steatosis is used for steatosis grading. Normal liver (grade 0) contains fat in <5% of hepatocytes; in grade 1, 2, 3 steatotic hepatocytes are present in <33%, 33–66% and >66% of hepatocytes, respectively, [41]. In the case of NASH histological diagnosis criteria include steatosis with hepatocellular (usually in the form of ballooning) and lobular inflammation [42]. There are three scoring systems that are currently used in grading the histological features of NAFLD/NASH, which are the Brunt system, the NAFLD Activity Score (NAS) and the Steatosis-Activity-Fibrosis (SAF) System [42,43,44,45]. Scoring in individual systems together with scored histological features are presented in Table 2, Table 3, Table 4 and Table 5.

## 5. Treatment Options of NAFLD

A considerable amount of research points out strong evidence between NASH and lifestyle modifications such as: weight loss, dietary changes and physical exercises. It has been proven that weight reduction by 5 to 10% in individuals with obesity can result with improvement in all features of NASH, including inflammation and fibrosis [46]. Dietary changes should include decrease in calorie intake, as well as changes in composition of a diet that includes reduction of carbohydrate intake (particularly simple carbohydrates, e.g., sweets, fruit juices, honey, fruits, flavored yoghurts), reduction of dietary fats with emphasis on saturated and trans fatty acids, increase in protein intake, ensuring supply of antioxidants, probiotics and prebiotics. Abstinence from alcohol is also recommended as a lifestyle intervention in NAFLD treatment [47]. However, it is very important to notice that implementing lifestyle modifications in patients with obesity can be problematic and usually does not bring the intended results. A study conducted by Dudekula et al. that aimed to find weight loss predictors in patients with obesity and NAFLD showed that 66% of research participants experienced weight reduction of less than 5% during the observation period. Weight loss between 5 to 10% was observed in 12.9% patients and reduction in body weight >10% was seen only in 6.9% of study participants [48]. Additionally, most individuals with obesity are more likely to regain weight in a short period of time [49]. The general idea of NAFLD treatment focuses on co-existing diseases such as obesity, dyslipidemia, insulin resistance and diabetes mellitus. 

According to the European Association for the Study of the Liver (EASL) guidelines, pharmacological therapy should be implemented in patients with progressive NASH (bridging fibrosis and cirrhosis); early stage NASH with high risk for disease progression (increased ALT, presence of metabolic syndrome and diabetes mellitus, age >50 years) and active NASH with high necroinflammatory activities [50]. Pharmacological therapy options for NAFLD include: antidiabetic drugs, drugs modifying lipid profile, anti-obesity drugs, vitamin supplementation and novel therapeutic treatment that includes interference with inflammatory, fibrotic and apoptotic pathways. Among antidiabetics drugs pioglitazone, glucagon-like-peptide (GLP-1) analogues and liraglutide were found to be effective in NAFLD/NASH treatment. Pioglitazone was shown to significantly improve steatosis and inflammation, together with systemic and adipose- tissue resistance in one-year observation in patients with T2DM [51]. Research conducted by Bril et al. confirmed reduction of liver fibrosis and increase in adipose tissues insulin sensitivity. However, the effect was significantly greater in patients with type 2 diabetes than in patients with prediabetes [52]. Liraglutide is a long-acting GLP-1 agonist that improves key metabolic risk factors: weight, body mass index and glucose level. Besides its metabolic improvement, liraglutide was found to significantly improve liver steatosis in NAFLD patients by downregulating the expression of inflammatory mediators in the TNF-α signaling pathway [53,54]. Additionally, liraglutide affects the renin-angiotensin system (RAS), which is overactivated during NAFLD. Liraglutide was found to down regulate the ACE/Ang II/AT1R axis and antagonizes hepatocellular steatosis [55].

In the case of metformin, which is commonly used in prediabetes and diabetes treatment, no strong evidence for histological response was found in NAFLD patients [56]. Despite the fact that metformin has no specific influence on liver histology, it is recommended in NAFLD/NASH patients with T2DM due to its pleiotropic effect including reduction in body mass, and decrease in ALT activity and improvement of cardiovascular system [57]. Furthermore, a recent animal study conducted by Brandt et al. suggests that metformin has a protective effect on the development of NAFLD, which results from a protection against intestinal barrier impairment, e.g., loss of tight junction proteins. Metformin also alters intestinal microbiota composition in the proximal small intestine, which has a beneficial effect on steatosis development [58].

Vitamin supplementation has been also found to have its role in NAFLD treatment. Vitamins with antioxidant properties, such as Vitamin C and E decrease the oxidative stress that is seen in patients with NAFLD and NASH. Additionally, Vitamin E has anti-inflammatory and anti-apoptotic properties that can retard the fibrosis process and prevent from cirrhosis by modulating inflammatory response and cellular proliferation [59]. It should be mentioned that supplementation of Vitamin E is recommended for patients with NASH and stage 2 fibrosis proven in biopsy and without a family history of prostate cancer, as it was proven that high daily dose of Vitamin E (≥400 IU per day) is associated with progression of prostate cancer [60].

Data about usage of weight-loss medication in NAFLD are very scarce in the available literature. To date, only Orlistat was found to contribute to improvement in hepatic fat content, as well as the activity of ALT and AST during at least 24 weeks of therapy [61]. It is thought that Orlistat may have a potential beneficial effect on NAFLD as it stimulates weight loss, however it is not clear whether it has an independent effect on liver function. Other weight-loss medications such as naltrexone, bupropion and topiramate have no evidence of usefulness in NAFLD treatment [62].

The use of statins in NAFLD treatment is still controversial. Undoubtedly, statins decrease the level of total cholesterol, low-density lipoprotein cholesterol (LDL-C) and triglycerides, and hence limit the cardiovascular risk [63]. In the study conducted by Hyogo et al., patients were treated with 10 mg atorvastatin daily. Researchers observed significant reduction in AST, ALT and GGT concentrations as well as decrease in NAFLD Activity Score (NAS), which includes steatosis, hepatocyte ballooning and lobular inflammation [64]. The use of statins among patients with NAFLD should be implemented with co-existing dyslipidemia, as its protective effect on the cardiovascular system outweighs other adverse events and low efficacy on hepatic histopathology [47].

Among novel therapeutic perspectives, farnesoid X receptor (FXR) agonist has been investigated. Obeticholic acid (OCA or 6α-ethyl chenodeoxycholic acid, initially known as INT-747) is an FXR agonist registered for the treatment of primary biliary cholangitis due to its anticholestatic and hepatoprotective properties [65]. Data from recently performed clinical trials prove that OCA is effective in patients with biopsy-proven NASH or NAFLD [66,67]. The primary endpoint of FLINT study was histological improvement in NAFLD activity score of at least 2 points, which was achieved in 45% of patients receiving 25 mg OCA daily [66]. A study conducted by Mudaliar et al. showed that the administration of 25 or 50 mg OCA daily increases insulin sensitivity and reduces markers of hepatic inflammation and fibrosis in patient with NAFLD and T2DM [67]. Another farnesoid X receptor agonist, cilofexor (GS-9674) is under investigation as monotherapy or in combination with an acetyl-CoA carboxylase inhibitor, firsocostat (GS-0976). The combination of these two drugs showed improvement in liver steatosis and stiffness and serum markers of hepatic fibrosis [68]. Peroxisome proliferator-activated receptor (PPAR)-γ agonists such as rosiglitazone and pioglitazone have been under investigation for potential effects in NAFLD/NASH patients. The use of pioglitazone in patients with biopsy-proven NASH improves liver function and decreases liver fat content. Cusi et al. conducted a placebo-controlled RCT of 101 adults with NASH and T2DM. They documented that 58% of patients assigned to pioglitazone group (45 mg once daily) achieved the primary outcome (reduction in NAFLD activity score of at least 2 points without worsening of fibrosis) and 51% had resolution of NASH. Pioglitazone treatment was also associated with improvement in individual histological scores, including the fibrosis score, reducing hepatic triglyceride content from 19% to 7%, and improving adipose tissue, hepatic, and muscle insulin sensitivity [69]. A Fatty Liver Improvement with Rosiglitazone Therapy (FLIRT) trial showed that rosiglitazone improved steatosis and normalized transaminase levels in 47% of patients. However, no effect on other histologic lesions was documented [70].

Some experimental studies have focused on the specific inhibition of the fibrosis process in liver with the use of an inhibitory antibody to lysyl oxidase-2 (LOXL-2). LOXL-2 up-regulation was noticed in patients with NAFLD and T2DM and LOXL-2 hepatic and circulating levels correlate with histological fibrosis progression [71]. LOXL-2 inhibition paves the way for macrophage-mediated collagen degradation in liver fibrosis. However, in two phase 2b trails of patients with bonding fibrosis due to nonalcoholic steatohepatitis, simtuzumab (monoclonal LOXL-2 antibody) was found to be ineffective in decreasing hepatic collagen content [72]. Additionally, compounds interfering with apoptotic pathways have been investigated as a treatment option for NAFLD/NASH. An example is selonsertib, which is an inhibitor of the apoptosis signal-regulating kinase 1 (ASK1), and plays a significant role in hepatocyte inflammation, injury and fibrosis. In a phase 2 trial, selonsertib appeared to improve liver fibrosis in a substantial proportion of patients with NASH and stage 2 or 3 fibrosis, suggesting its potential use in NAFLD pharmacological therapy [73]. However, results from randomized phase III STELLAR trials did not show evidence that selonsertib reduces fibrosis in patients with NASH and advanced liver scarring [74].

## 6. Bariatric Surgery and NAFLD

Bariatric surgery aims not only to achieve considerable, long-term weight loss but also to improve the course of obesity-related diseases such as T2DM, hypertension, dyslipidemia, obstructive sleep apnea. It also reduces the risk of cardiovascular diseases such as myocardial infarction and ischemic stroke and decreases overall mortality [75,76,77]. A meta-analysis conducted by Sutanto et al. showed significant reduction in the incidence of major adverse cardiovascular events in bariatric surgery group as compared to the no-surgery group (OR = 0.49; 95% CI 0.40–0.60; *p* < 0.00001; I2 = 93%) [78]. Among recently available surgical methods, Roux-en-Y gastric bypass (RYGB) and laparoscopic sleeve gastrectomy (LSG) are the most commonly performed worldwide. A study conducted by Mummadi et al. summarized 15 studies with 766 paired liver biopsies. Their investigation showed the pooled proportion of patients with improvement or resolution in steatosis was 91.6% (95% confidence interval (CI), 82.4–97.6%), in steatohepatitis was 81.3% (95% CI, 61.9–94.9%), in fibrosis was 65.5% (95% CI, 38.2–88.1%), and for complete resolution of NASH was 69.5% (95% CI, 42.4–90.8%) after bariatric surgery [79]. The Swedish Obese Subjects (SOS) study showed reduction in both ALT and AST values after bariatric surgery in both short and long-term observation (2 and 10-year follow-up) [80].

NAFLD is closely associated with obesity, T2DM and other features of metabolic syndrome. All mechanisms involved in improving obesity and T2DM that appear after bariatric surgery seem to have a crucial role in amelioration or resolution of NAFLD. Weight reduction due to bariatric surgery causes inflammatory changes in patients with obesity. Klein et al. showed that gastric bypass procedure decreases the hepatic expression of factors involved in the progression of liver inflammation (macrophage chemoattractant protein 1 (MCP-1), and interleukin (IL-8)) and fibrogenesis (transforming growth factor-β1 (TGF-β1), tissue inhibitor of metalloproteinase 1 (TIMP-1), α-smooth muscle actin (α-SMA), and collagen-α1(I)) [81]. Cazzo et al. showed a significant decrease in mean NAFLD fibrosis score after RYGB and resolution rate of 55% of severe fibrosis in 12-month observation [82]. Moreover, RYGB contributes to significant reduction in NAFLD activity score, steatosis, inflammation and liver ballooning during 1-year observation [83,84].

LSG is also considered to improve the course of NAFLD. Nobili et al. showed reduced activation of local cellular compartments (hepatic progenitor cells, hepatic stellated cells, macrophages) induced by LSG, which led to the improvement in NAFLD Activity Score and liver fibrosis [85]. A study conducted by Cabré et al. proved that the histology and liver function of patients with morbid obesity significantly improved after LSG due to mechanisms involved in the reduction of oxidative stress and inflammation. They observed significant reduction in the hepatic immunochemical expression of oxidation, inflammation and fibrosis markers such as: PON-1, 4-hydroxy-2-nonenal, CD68, chemokine ligand 2 (CCL2), C-C chemokine receptor type 2 (CCR2), TNF-α, and galectin-3 between baseline liver tissue and 12 months after LSG [86]. Weight loss induced by LSG leads to the improvement in liver histology in terms of steatosis, liver fibrosis, lobular inflammation and hepatocyte ballooning. In a study conducted by Salman et al., among 81 patients undergoing LSG, 9 (11.1%) showed no steatosis at the end of 18-month follow-up, 25 (30.9%) showed no hepatocyte ballooning, 37 (45.7%) showed no lobular inflammation, and 33 (40.7%) showed complete absence of fibrosis. The above-mentioned study also showed significant improvement in postoperative liver function tests (AST, ALT, GGTP). An 18-month observation also revealed an increase in adiponectin levels and a reduction in serum levels of leptin and resistin, when compared to presurgical values. The above-mentioned data prove that both LSG and RYGB are significant surgical methods for NAFLD/NASH treatment [87].

As presented above, bariatric surgery provides proven NAFLD amelioration; however, the remaining question is whether RYGB or LSG is more effective. A systematic review and meta-analysis performed by Baldwin et al. compared RYGB and LSG using 4 separate criteria: AST and ALT concentration, NAFLD activity score and NAFLD fibrosis score. Patients undergoing both procedures showed significant reduction in AST and ALT values. Head-to-head comparison of AST mean differences trended toward LSG, but it was statistically non-significant. This study failed to show superiority between RYGB and LSG in ameliorating NAFLD [88]. Cherla et al. also proved the normalization of the liver function test by the end of the first postoperative year; however, they did not find significant differences between the SG and RYGB groups [89]. A meta-analysis performed by Silva et al. showed that RYGB patients achieve significant reduction of steatohepatitis and fibrosis, while patients undergoing LSG presented significant reduction only of steatohepatitis. According to their study, the NAFLD Activity Score significantly improved after both procedures and no differences were found between LSG and RYGB regarding histopathological changes [90]. A study conducted by Pedersen et al. showed that NAS reduced significantly in both RYGB and LSG patients 12-months after the surgery. However, RYGB patients had significantly more reduced (*p* = 0.007) liver steatosis (−0.91 (95% CI −1.47–−1.2) than SG patients (−0.33 (95% CI −0.54–−0.13) and greater improvement in the plasma lipid profile [83]. Luo at el. investigated liver volume and fat density in MRI in patients undergoing bariatric surgery. Their study showed that RYGB patients achieved higher weight loss and higher BMI loss when compared to the LSG group. However, the percentage decrease in liver volume and MRI-PDFF did not differ significantly between groups [91].

Despite the significant role of bariatric surgery in the treatment of NAFLD, there are some patients that will develop new or worsened features of NAFLD after bariatric procedure. The meta-analysis performed by Lee et al. showed that 12% of patients experienced development or worsening of NAFLD (95% CI, 5–20%) [92]. A 5-year prospective study performed by Mathurin et al. showed that 19.8% of patients experienced fibrosis progression 5 years after bariatric surgery for unknown reason [93]. Aggravation of NAFLD after bariatric procedure should be kept in mind when qualifying patients for bariatric surgery.

## 7. Conclusions

The current evidence suggests that bariatric/metabolic surgery for patients with morbid obesity leads to improvement or resolution of NAFLD/NASH in terms of steatosis, hepatic inflammation and fibrosis. Although the results of available cohort research are satisfying, they have not been proved in clinical randomized trails. Further, long-term studies are still needed to confirm the recommendation of bariatric surgery as a treatment option for NAFLD.

## Figures and Tables

**Table 1:** Noninvasive assessment of liver fibrosis based on biochemical parameters.

Name of Scoring System	Used Measures
NAFLD fibrosis score (NFS)	Age, blood glucose level, BMI, platelet count, albumin, AST/ALT ratio
APRI score	aspartate aminotransferase to platelet ratio index
BAAT score	BMI, age, ALT, triglyceride level
BARD score	BMI, AST/ALT ratio, presence/absence of diabetes
Enhanced liver fibrosis (ELF) index	Plasma level of hyaluronic acid (HA), tissue inhibitor of metalloproteinase (TIMP-1), procollagen III amino terminal peptide (PIIINP)
Hepascore	Bilirubin, gamma-glutamyl transpeptidase (γ-GTP), α2-macroglobulin, hyaluronic acid levels
FIBROSpect	hyaluronic acid, TIMP-1 and α2-macroglobulin
Fibrometer	prothrombin index, platelet count, AST, urea, α2-macroglobulin, hyaluronic acid
NashTest	age, sex, height, weight, serum triglycerides, cholesterol, α2-macroglobulin, apolipoprotein A1, haptoglobin, γ-GTP, ALT, AST, total bilirubin

**Table 2:** Brunt system to grade NASH activity.

**Grade**	**Steatosis** 1: ≤33% 2: 33–66% 3: ≥66%	**Ballooning** (Zonal Location and Severity Recorded)	**Inflammation**	
			L-Lobular (0–3) 0: Absent 1: <2 foci/20× field 2: 2–4 foci/20× field 3: >4 foci/20× field	P-Portal (0–3) 0: Absent 1: Mild 2: Moderate 3: Severe
**Grade 1 (mild)**	1–2	Minimal, zone 3	L = 1–2	P = 0–1
**Grade 2 (moderate)**	2–3	Present, zone 3	L = 2	P = 1–2
**Grade 3 (severe)**	2–3	Marked, predominantly zone 3	L = 3	P = 1–2

**Table 3:** Brunt system for staging NASH fibrosis.

Stage	Zone 3, Sinusoidal	Portal Based	Bridging	Cirrhosis
**1**	Focal or extensive	0	0	0
**2**	Focal or extensive	Focal or extensive	0	0
**3**	Bridging septa	Bridging septa	+	0
**4**	±	±	Extensive	+

**Table 4:** The NAFLD Activity Score.

Steatosis Grade (S)	Lobular Inflammation (L)	Hepatocyte Ballooning (B)
0: <5%	0: none	0: none
1: 5–33%	1: <2 foci/20× field	1: mild, few ballooned cells
2: 34–66%	2: 2–4 foci/20× field	2: moderate-marked, many ballooned cells
3: >66%	3: >4 foci/20× field	
**Fibrosis** (evaluated with Masson trichrome stain)		
0		None
1a		Mild zone 3 sinusoidal fibrosis (trichrome stain to be identified)
1b		Moderate zone 3 sinusoidal fibrosis (could be detected on H&E examination)
1c		Portal fibrosis only
2		Zone 3 sinusoidal fibrosis and periportal fibrosis
3		Bridging fibrosis
4		Cirrhosis

**Table 5:** Steatosis-Activity-Fibrosis (SAF) scoring system of NAFLD.

Steatosis Grade (S): 0–3 (Based on Percentage of Hepatocytes with Large and/or Medium Size Intracytoplasmic Lipid)	Lobular Inflammation: 0–2	Hepatocyte Ballooning: 0–2	Activity Grade (A): 0–4 (Sum of Score for Ballooning and Lobular Inflammation)	Fibrosis Stage (F)
S0: <5%	0: none	0: none	A1 (A = 1): mild activity	F0: no significant fibrosis
S1: 5–33%	1: ≤2 foci/20× field	1: cluster of rounded hepatocytes with pale/reticulated cytoplasm	A2 (A = 2): moderate activity	F1: 1a mild zone 3 sinusoidal fibrosis 1b moderate zone 3 sinusoidal fibrosis 1c portal fibrosis only
S2: 34–66%	2: >2 foci/20× field	2: same as 1 with enlarged hepatocytes (more than twice of normal size)	A3 and A4 (A > 2): severe activity	F2: zone 3 sinusoidal fibrosis with periportal fibrosis
S3: >66%				F3: bridging fibrosis
				F4: cirrhosis

## Data Availability

Not applicable.
